# The Least Nodal Disease Burden Defines the Minimum Number of Nodes Retrieved for Esophageal Squamous Cell Carcinoma

**DOI:** 10.3389/fonc.2022.764227

**Published:** 2022-03-03

**Authors:** Hanlu Zhang, Xiuji Yan, Yu-Shang Yang, Hong Yang, Yong Yuan, Dong Tian, Yin Li, Zhi-Yong Wu, Yun Wang, Jian-Hua Fu, Long-Qi Chen

**Affiliations:** ^1^ Department of Thoracic Surgery, West China Hospital of Sichuan University, Chengdu, China; ^2^ Department of Plastic and Burns Surgery, West China Hospital of Sichuan University, Chengdu, China; ^3^ Department of Thoracic Oncology, Sun Yatsen University Cancer Center, Guangzhou, China; ^4^ Department of Thoracic Surgery, Affiliated Hospital of North Sichuan Medical College, Nangchong, China; ^5^ Department of Thoracic Surgery, Cancer Hospital Chinese Academy of Medical Sciences, Beijing, China; ^6^ Department of Oncology Surgery, Affiliated Shantou Hospital of Sun Yat-sen University, Shantou, China

**Keywords:** esophageal squamous cell carcinoma, surgery, lymph node dissection, lymph node count, prognosis

## Abstract

**Background:**

Clinically, a single positive lymph node (SPLN) should indicate the least nodal disease burden in node-positive patients with esophageal squamous cell carcinoma (ESCC) and may also be used to define the minimum number of examined lymph nodes (NELNs) in ESCC patients.

**Methods:**

Data from three Chinese cohorts of 2448 ESCC patients who underwent esophagectomy between 2008 and 2012 were retrospectively analyzed. Based on lymph node status, patients were divided into two groups: N0 ESCC and SPLN ESCC. A Cox proportional hazards regression model was used to determine the minimum NELNs retrieved to maximize survival for ESCC patients with localized lymph node involvement. The results were then validated externally in the SEER database.

**Results:**

A total of 1866 patients were pathologically diagnosed with N0 ESCC, and 582 patients were diagnosed with SPLN ESCC. The overall survival rate of patients with N0 ESCC was significantly better than that of patients with SPLN ESCC (HR 1.88, 95% CI 1.64-2.13, *P*<0.001), but no significant difference was found between SPLN ESCC patients with ≥ 20 lymph nodes harvested and N0 ESCC patients (HR 1.20, 95% CI 0.95-1.52, *P*=0.13). Analysis of patients selected from the SEER database showed the same trend, and no significant difference was observed between N0 ESCC patients and SPLN ESCC patients with ≥ 20 lymph nodes retrieved (HR: 1.02, 95% CI 0.72-1.43, *P*=0.92).

**Conclusions:**

A minimum of 20 lymph nodes retrieved should be introduced as a quality indicator for ESCC patients with localized lymph node involvement.

## Introduction

Esophageal carcinoma was the sixth leading cause of cancer-related mortality and the seventh most common cancer worldwide in 2018 (1). Squamous cell carcinoma is the predominant pathological type. It is an extremely aggressive gastrointestinal cancer with a poor prognosis. Even after radical surgical resection, the recurrence rate ranges from 43 to 53%, and the 5-year overall survival (OS) rate ranges from 15-20% (2). Lymph node metastasis is the most common mode of tumor spread and is an important prognostic factor (3, 4).

Esophagectomy with radical lymphadenectomy remains the standard treatment for operable esophageal squamous cell carcinoma (ESCC) patients. The possible presence of occult tumor dissemination is the rationale for radical systematic lymphadenectomy. The number of retrieved lymph nodes is regarded as a quality indicator for ESCC surgery (5, 6). Theoretically, the greater the extent of lymphadenectomy is, the more similar the survival outcomes between node-negative and node-positive ESCC patients. The authors speculate that a certain number of lymph nodes retrieved may allow SPLN ESCC patients to experience the same survival benefits as N0 ESCC patients; if so, this cutoff point should be defined as the minimum requirement for an adequate extent of lymphadenectomy. The aim of this study was to define the minimum number of examined lymph nodes (NELNs) harvested for ESCC patients with limited lymph node involvement.

## Patients and Methods

### Patients in the Training Cohort

Between 2008 and 2012, ESCC patients who underwent radical surgical resection at three high-volume centers in China (West China Hospital, Shantou University Medical College and Sun Yat-sen University Cancer Center) were enrolled in this retrospective analysis. The analysis was limited to patients with negative lymph nodes (N0) and a single positive lymph node (SPLN) based on the postoperative histopathological examination. The exclusion criteria were as follows: (1) patients with nonsquamous cell carcinoma; (2) patients receiving neoadjuvant therapy; (3) patients with cervical esophageal cancer; and (4) patients with surgical-related mortality (defined as death occurring within 1 month of the operation). All patients underwent subtotal esophagectomy with two-field lymphadenectomy, including the Sweet, Ivor-Lewis or McKeown approach depending on the location and extent of the tumor. The study protocol was approved by our institutional review board (2019-441). Informed consent was waived because of the retrospective nature of the study.

Lymph nodes were identified and detached from the operative specimen by the surgeons during surgery. Lymph node metastasis was assessed by expert pathologists. The patients were followed up every 3 months for the first 2 years after surgery and then every 6 months for the subsequent 3 years. Thereafter, follow-up visits were conducted annually until death or June 2016. The primary outcome was OS. OS was defined as the time from surgery to the date of death or the last clinical visit. Patients who were alive at the last follow-up were censored for OS.

### Patients in the Validation Cohort

ESCC patients who underwent esophagectomy and lymphadenectomy between 2004 and 2016 were selected from the Surveillance, Epidemiologic, and End Results (SEER) database to perform external validation. The exclusion criteria were as follows: patients with incomplete data, patients with nonsquamous cell carcinoma, patients who died within 1 month of the operation, and patients with distant metastasis. Ultimately, a total of 1316 ESCC patients from the SEER database who fulfilled the inclusion and exclusion criteria were eligible for the analysis.

### Statistical Analysis

Continuous variables are described as the mean ± standard deviation (SD), and categorical variables are described as the frequency (%). Distributions’ normality of the variables was checked by Lilliefors-test. The chi-square test or Fisher’s exact test was used to compare the distribution of categorical variables between groups. Continuous variables were analyzed using Student’s t test or the Wilcoxon rank-sum test. Univariate analysis was performed to examine the association between potential predictors and survival. Factors with *P* < 0.25 in the univariate analysis and believed to be associated with cancer-related deaths were entered into a multivariate Cox proportional hazards regression model. A backward stepwise elimination of variables was used to construct the final model. A two-sided *P* value < 0.05 was considered statistically significant.

Patients in this study were divided into two groups: ESCC patients with no lymph node metastasis (N0) and ESCC patients with an SPLN. N0 patients were defined as the reference group and were compared with SPLN patients with various numbers of harvested lymph nodes. To define the minimum NELNs that need to be removed, a multivariate analysis was performed and adjusted for potential risk factors. A certain number of lymph nodes was regarded as the minimum NELNs that needs to be removed when the OS analysis of SPLN patients with a certain number of lymph nodes retrieved showed no significant difference compared with that of N0 ESCC patients. We did the Kaplan-Meier survival curves by the log-rank test, which was used to analyze the differences between the curves. Data analysis was performed with SPSS version 24.0 (SPSS Inc., Chicago, IL, United States).

## Results

### Patient Characteristics

A total of 2448 ESCC patients in the training cohort were included in this study. Among them, 582 patients were diagnosed with an SPLN. And 1866 patients were diagnosed with N0 who were defined as the reference group. A total of 1316 patients from the SEER database fulfilled the criteria and were further analyzed in this study as the validation cohort. Among them, 1079 patients were diagnosed with N0 ESCC, and 237 patients were diagnosed with SPLN ESCC. The demographics and clinical characteristics of the training and validation cohorts are shown in **Table 1**.

**Table 1:** Demographics and clinical characteristics of the two cohorts.

Variables	Training cohort			Validation cohort		
	N0 (n = 1866)	SPLN (n = 582)	*P* value	N0 (n = 1079)	SPLN (n = 237)	*P* value
Age (Mean ± SD, years)	59.4 ± 8.5	59.5 ± 8.8	0.87	63.8 ± 9.6	63.5 ± 9.4	0.73
Gender (n, %)			0.02			0.33
Male	1417 (75.9%)	469 (80.6%)		651 (60.3%)	151 (63.7%)	
Female	449 (24.1%)	113 (19.4%)		428 (39.7%)	86 (36.3%)	
pTNM stage (n, %)			< 0.001			< 0.001
I/II	1721 (92.2%)	147 (25.3%)		858 (79.5%)	89 (37.6%)	
III	145 (7.8%)	435 (74.7%)		221 (20.5%)	148 (62.4%)	
NELN (mean ± SD)	15.6 ± 9.4	16.6 ± 8.7	0.02	14.4 ± 10.6	14.8 ± 10.7	0.71
pT stage (n, %)			< 0.001			< 0.001
pT1/pT2	762 (40.8%)	149 (25.6%)		560 (51.9%)	88 (37.1%)	
pT3/pT4	1104 (59.2%)	433 (74.4%)		519 (48.1%)	149 (62.9%)	
Differentiation (n, %)			0.06			0.006
Well/Moderate	1205 (64.6%)	351 (60.3%)		611 (56.6%)	111 (46.8%)	
Poor	661 (35.4%)	231 (39.7%)		468 (43.4%)	126 (53.2%)	

### Minimum NELNs for ESCC Patients With Localized Lymph Node Involvement

**Figure 1:**
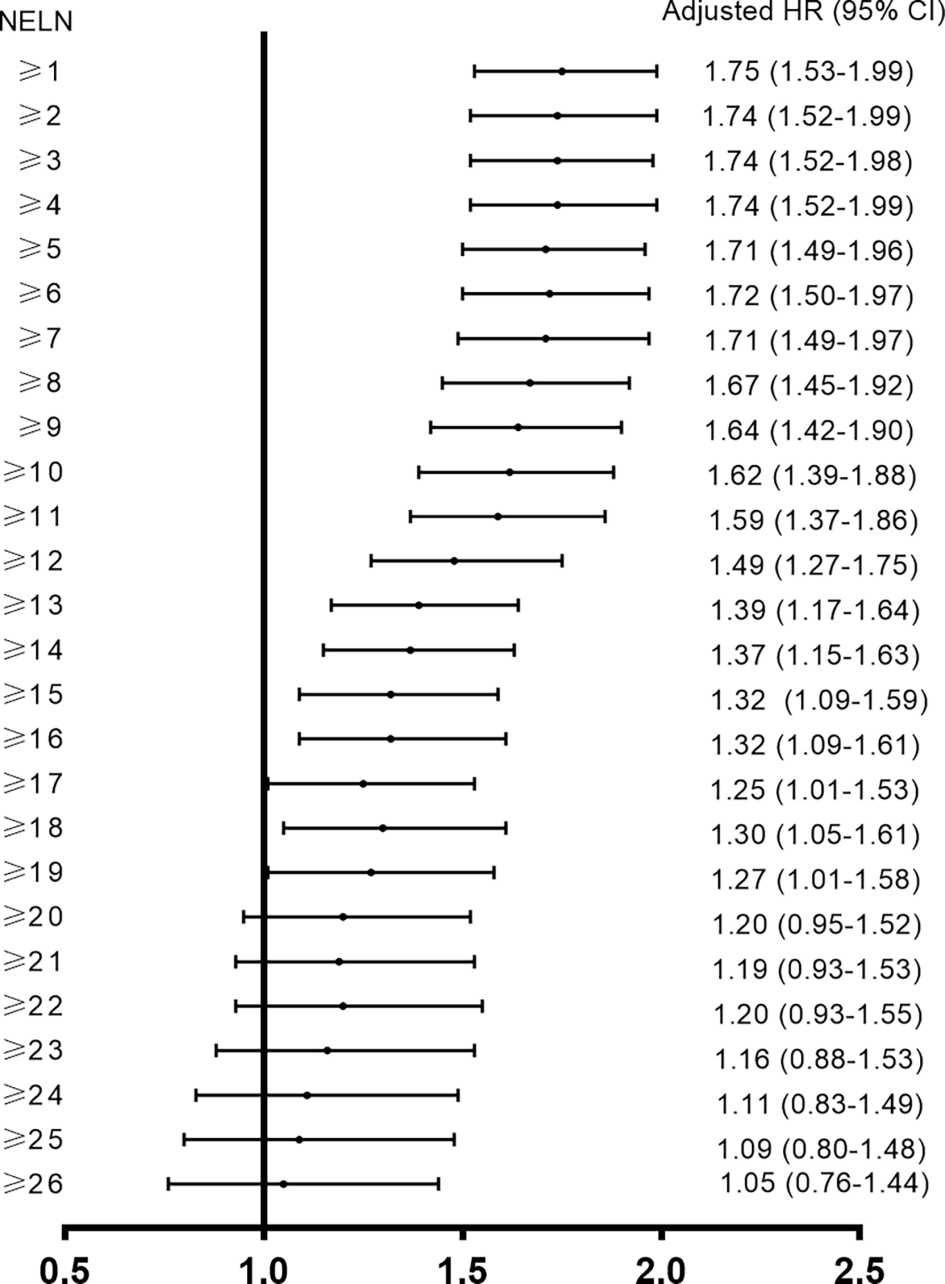
Adjusted estimated hazard ratios for SPLN ESCC patients with different NELN (Reference group: N0 ESCC patients).

**Figure 2:**
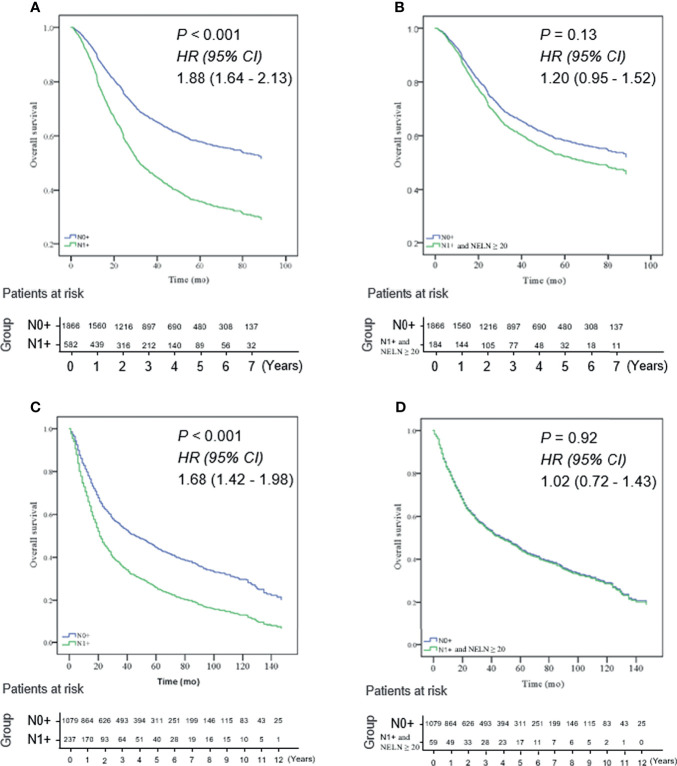
**(A)** N0 ESCC patients had a significantly better OS rate than those with SPLN (N1 +) ESCC in the training cohort (HR 1.88, 95% CI 1.64 - 2.13, *P* < 0.001); **(B)** no significant difference was found between SPLN ESCC patients with ≥ 20 lymph nodes harvested and N0 ESCC patients in the training cohort (HR 1.20, 95% CI 0.95 - 1.52, *P* = 0.13); **(C)** OS of N0 ESCC patients was significantly better than that of SPLN ESCC patients in the validation cohort (HR 1.68, 95% CI 1.42 - 1.98, *P* < 0.001); **(D)** OS rate of SPLN ESCC patients with ≥ 20 lymph nodes harvested was not significantly different from that of N0 ESCC patients in the validation cohort (HR: 1.02, 95% CI 0.72 - 1.43, *P* = 0.92).

To maximize survival, a number of nodes need to be removed for ESCC patients with localized lymph node involvement. First, univariate analysis was performed. Age, sex, pT stage, tumor location, tumor differentiation and adjuvant therapy were identified as potential prognostic factors of ESCC patients (**Table 2**). Then, a Cox proportional hazards regression model for OS was generated between N0 and SPLN ESCC patients and adjusted for age, sex, pT stage, tumor location, tumor differentiation and adjuvant therapy (**Table 2**). N0 patients were defined as the reference group and were compared with SPLN patients with various numbers of harvested lymph nodes. Adjusted estimated hazard ratios for SPLN ESCC patients with different NELNs were shown in **Figure 1**. Patients with N0 ESCC had a significantly better OS rate than those with SPLN ESCC (HR 1.88, 95% CI 1.64-2.13, *P*<0.001, **Figure 2A**). However, no significant difference was found between SPLN ESCC patients with ≥ 20 lymph nodes harvested and N0 ESCC patients (HR 1.20, 95% CI 0.95-1.52, *P*=0.13, **Figure 2B**). Therefore, at least 20 lymph nodes must be resected for SPLN ESCC patients to maximize survival. Since SPLN ESCC indicates the least nodal disease burden, we speculate that 20 is the minimum number of lymph nodes that need to be dissected for ESCC patients with localized lymph node involvement.

**Table 2:** Univariate and multivariate cox regression analysis of independent prognostic factors for ESCC patients.

Variables	Univariate analysis				Multivariate analysis			
	Exp (B)	95% CI		*P* value	Exp (B)	95% CI		*P* value
		Lower	Upper			Lower	Upper	
Age	1.12	0.99	1.27	0.07	1.09	0.96	1.23	0.20
Gender	0.91	0.79	1.06	0.23	0.96	0.83	1.11	0.58
pT	1.94	1.69	2.23	< 0.001	1.92	1.66	2.21	< 0.001
Differentiation	1.22	1.07	1.38	0.002	1.28	1.13	1.46	< 0.001
Tumor location	0.90	0.77	1.04	0.13	0.85	0.74	0.99	0.03
Adjuvant therapy	1.00	0.87	1.14	0.98	0.86	0.75	0.98	0.03
LN metastasis	1.88	1.64	2.14	< 0.001	1.75	1.53	1.99	< 0.001

There were 674 patients in the training cohort and 157 patients in the validation cohort had 20 or more lymph node dissection. Then, we validated whether the cutoff of 20 lymph nodes was also suitable for ESCC patients selected from the SEER database. Intriguingly, analysis of patients selected from the SEER database showed the same result: the OS of N0 ESCC patients was significantly better than that of SPLN ESCC patients (HR 1.68, 95% CI 1.42-1.98, *P*<0.001, **Figure 2C**). However, the OS rate of SPLN ESCC patients with ≥ 20 lymph nodes harvested was not significantly different from that of N0 ESCC patients (HR: 1.02, 95% CI 0.72-1.43, *P*=0.92, **Figure 2D**), which validated the results of the multicenter cohort described above.

**Figure 3:**
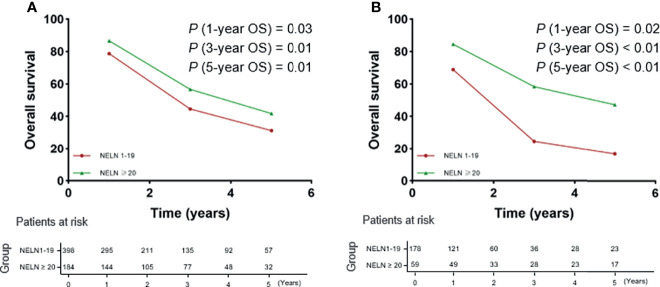
1-year, 3-year and 5-year OS rates of SPLN ESCC patients with 1-19 lymph nodes retrieved (Red line) and ≥ 20 lymph nodes retrieved (Green line). **(A)** the training cohort; **(B)** the validation cohort.

**Figure 3** shows OS according to the number of lymph nodes harvested. In detail, for SPLN ESCC patients with 0 to 19 lymph nodes retrieved, the 1-year, 3-year and 5-year OS rates were 78.7%, 61.2% and 31.3%, respectively. For SPLN ESCC patients with more than 20 lymph nodes retrieved, the 1-year, 3-year and 5-year OS rates were 86.6%, 56.7% and 41.8%, respectively (1-year the HRs with 95%CI was 0.59, 0.37-0.93; 3-year the HRs with 95%CI was 0.69, 0.53-0.91 and 3-year the HRs with 95%CI was 0.73, 0.57-0.94, **Figure 3A**). A trend of improved OS was also observed for SPLN ESCC patients from the SEER database (1-year the HRs with 95%CI was 0.43, 0.21-0.87; 3-year the HRs with 95%CI was 0.40, 0.25-0.62 and 3-year the HRs with 95%CI was 0.42, 0.27-0.63, **Figure 3B**). Therefore, more lymph nodes harvested during surgical resection predicts improved OS for SPLN ESCC patients.

## Comment

The presence of lymph node metastasis affects the prognosis of ESCC patients (7). The latest UICC/AJCC staging system proposed pN classification based on the number of metastatic lymph nodes (8). Nodal disease burden includes not only the positive lymph nodes examined microscopically but also the occult tumor dissemination. Since the number of metastatic nodes cannot be assessed precisely before or during surgery, extensive lymphadenectomy could eradicate both overt metastasis and occult lymph node metastasis, which may result in a better prognosis and reduce stage migration (9–11). However, a greater number of retrieved lymph nodes may increase the risk of intraoperative and postoperative morbidity and the mortality rate (12). The optimal extent of lymph node dissection for ESCC patients during esophagectomy has not been clearly defined (10, 13–15). In addition, since the increasing percentage of older patients may result in more patients considered marginal candidates for esophageal resection and not all of the patients may have the physiologic reserve to receive timely postoperative adjuvant therapy after surgery (16, 17), adequate resection is needed to reduce locoregional failure. Therefore, the optimal extent of lymphadenectomy needs to be determined for ESCC patients undergoing radical resection.

Lymphadenectomy strategy may be different for esophageal cancer patients with different lymph node status. According to Peyre et al. (18), the probability of systemic disease exceeds 50% when 3 or more nodes are involved and approaches 100% when 8 or more nodes are involved. Dr. Omloo and Hulscher et al. demonstrated that an extended lymphadenectomy did not provide survival benefit for esophageal cancer patients with more than 8 positive lymph nodes (19, 20). Therefore, a survival benefit may not be achieved through extended lymphadenectomy for patients with an advanced pN stage, and multimodality treatment may be needed (15). Regarding ESCC patients with localized lymph node involvement (especially SPLN ESCC patients), we speculate that they are still at a stage where they can be cured by surgery. In comparison with ESCC patients with two or more positive lymph nodes, SPLN ESCC patients may need the minimum NELNs harvested to maximize survival. Accordingly, we selected SPLN ESCC patients to investigate the minimum NELNs to maximize OS for ESCC patients with limited lymph node involvement.

If clinically positive lymph nodes are suspected before surgery, neoadjuvant chemoradiation therapy followed by surgery is routinely recommended to maximize survival (12, 21, 22). CT, endoscopic ultrasound and positron emission tomography are the most common preoperative work-ups for ESCC patients. However, they are not precise enough to predict lymph node metastasis (23), and undetected nodal disease is usually encountered with upfront esophagectomy (24). Esophagectomy and sufficient lymph node resection are essential for accurate staging and improving survival, especially for ESCC patients with localized lymph node involvement.

Cut-point survival analysis is usually used to investigate the optimal cutoff point of the minimum NELNs to maximize survival for cancer patients (25, 26). However, our study is markedly different from previous studies. We utilized a novel method to investigate the minimum number of lymph nodes examined for patients with esophageal squamous cell carcinoma. We regarded ESCC node-negative patients as the reference group to investigate the minimum NELNs for overt metastasis and micrometastasis to be eradicated in SPLN patients. Ultimately, 20 lymph nodes was determined to be the minimum NELNs to maximize survival for SPLN ESCC patients; therefore, a total of 20 lymph nodes retrieved may be the minimum number of lymph nodes to eliminate nodal disease. Since SPLN ESCC patients have a lower potential for lymph node metastasis than other pN+ ESCC patients, we speculate that 20 might be the minimum NELNs for ESCC patients with a relatively low nodal disease burden.

It is important to externally validate the results of our study in other clinical settings, so we retrieved data from the SEER database for further analysis. Because the patients in the training and validation cohorts lived in two parts of the world, they had completely different clinicopathological characteristics. Intriguingly, patients selected from the SEER database showed the same trend as those in the base cohort. The results obtained from the validation cohort strengthen our research and indicate that our results have good universality. In clinical practice of upfront surgery for patients with ESCC, at least 20 lymph nodes should be resected and it may be a quality indicator.

The result of this multicenter study was validated externally by using data from the SEER database, but it has some limitations. This was a retrospective study, and no subgroup analysis was conducted on each pT stage because of the small number of SPLN patients. ESCC patients with negative nodes were defined as the reference group, but a number of patients with false negatives may have been included in the group. Therefore, 20 may be the minimum NELNs for pN+ ESCC patients with limited lymph node involvement. In addition, patients who received neoadjuvant were not included in this study. The present result may be applicable only to operable ESCC patients undergoing upfront surgery. Both neoadjuvant chemoradiotherapy and extensive lymph node dissection improve locoregional tumor control (27), so patients receiving neoadjuvant chemoradiotherapy might need extended resection, and a different lymph node dissection strategy may be needed. Further studies should investigate the minimum NELNs for ESCC patients receiving neoadjuvant chemoradiotherapy.

## Data Availability Statement

The raw data supporting the conclusions of this article will be made available by the authors, without undue reservation.

## Ethics Statement

The institutional review boards of each center approved this study. The ethics committee waived the requirement of written informed consent for participation.

## Author Contributions

L-QC and J-HF contributed the idea and design. HZ and XY collected the data. All authors contributed to the article and approved the submitted version.

## Funding

This study was funded by 1•3•5 project for disciplines of excellence–Clinical Research Incubation Project, West China Hospital, Sichuan University (2018HXFH020), Chengdu Science & Technology Bureau Grant (2019-GH02-00080-HZ), Sichuan Science and Technology Program (2021YFS0222).

## Conflict of Interest

The authors declare that the research was conducted in the absence of any commercial or financial relationships that could be construed as a potential conflict of interest.

## Publisher’s Note

All claims expressed in this article are solely those of the authors and do not necessarily represent those of their affiliated organizations, or those of the publisher, the editors and the reviewers. Any product that may be evaluated in this article, or claim that may be made by its manufacturer, is not guaranteed or endorsed by the publisher.
